# The Mammalian Septin Interactome

**DOI:** 10.3389/fcell.2017.00003

**Published:** 2017-02-07

**Authors:** Katharina Neubauer, Barbara Zieger

**Affiliations:** Division of Pediatric Hematology and Oncology, Department of Pediatrics and Adolescent Medicine, Faculty of Medicine, Medical Center–University of FreiburgFreiburg, Germany

**Keywords:** septins, septin-multimers, septin-interacting proteins, platelets, human endothelial cells

## Abstract

Septins are GTP-binding and membrane-interacting proteins with a highly conserved domain structure involved in various cellular processes, including cytoskeleton organization, cytokinesis, and membrane dynamics. To date, 13 different septin genes have been identified in mammals (*SEPT1* to *SEPT12* and *SEPT14*), which can be classified into four distinct subgroups based on the sequence homology of their domain structure (SEPT2, SEPT3, SEPT6, and SEPT7 subgroup). The family members of these subgroups have a strong affinity for other septins and form apolar tri-, hexa-, or octameric complexes consisting of multiple septin polypeptides. The first characterized core complex is the hetero-trimer SEPT2-6-7. Within these complexes single septins can be exchanged in a subgroup-specific manner. Hexamers contain SEPT2 and SEPT6 subgroup members and SEPT7 in two copies each whereas the octamers additionally comprise two SEPT9 subgroup septins. The various isoforms seem to determine the function and regulation of the septin complex. Septins self-assemble into higher-order structures, including filaments and rings in orders, which are typical for different cell types. Misregulation of septins leads to human diseases such as neurodegenerative and bleeding disorders. In non-dividing cells such as neuronal tissue and platelets septins have been associated with exocytosis. However, many mechanistic details and roles attributed to septins are poorly understood. We describe here some important mammalian septin interactions with a special focus on the clinically relevant septin interactions.

## Introduction

**Figure 1:**
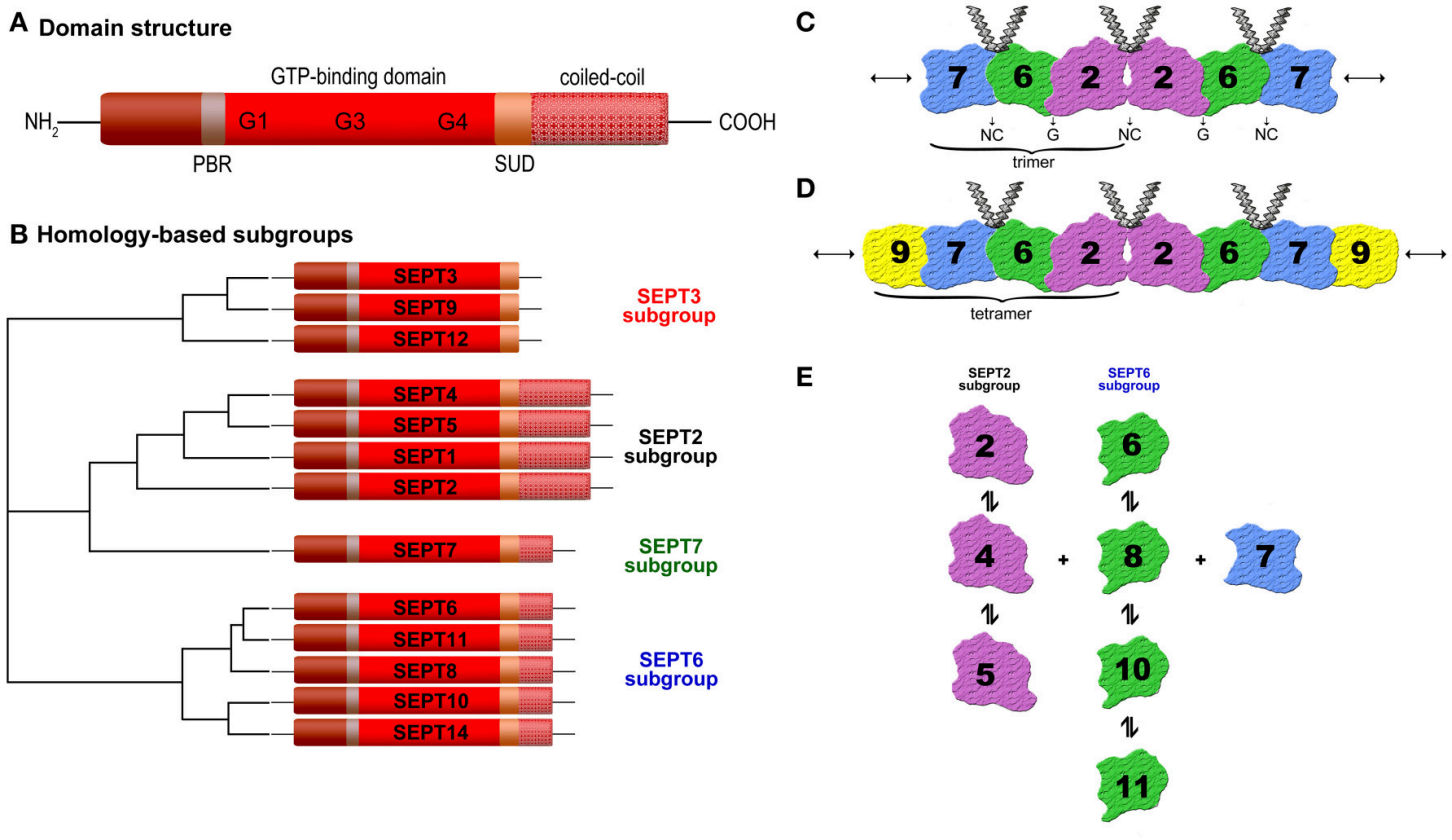
**(A)** Schematic septin domain structure. Septins share a conserved GTP-binding domain, a phosphoinosite-binding polybasic region (PBR), a septin unique domain (SUD), and most of them one or more coiled-coil domains. The length and amino acid sequence of the N- and C-terminus vary (according to Trimble, 1999). **(B)** Homology-based subgroups. The 13 human septins (SEPT1 to SEPT12 and SEPT14) are classified into four subgroups (SEPT3, SEPT2, SEPT7, and SEPT6) based on sequence homology and coiled-coil domains (Macara et al., 2002; Kinoshita, 2003b). **(C)** Structure of the SEPT2-6-7 complex. Two copies of each septin are symmetrically arranged (SEPT7-6-2-2-6-7) to generate a hexamer by alternating N- and C-termini (NC) and G-interface (GTP-binding domain) (Sirajuddin et al., 2007). **(D)** Structure of the SEPT2-6-7-9 complex (Sandrock et al., 2011; Kim et al., 2011). **(E)** Binding preferences of individual septins to other septins (Sandrock et al., 2011).

Septins (SEPTs) have been originally discovered in *Saccharomyces cerevisiae* as a family of proteins associated with cytokinesis and cell morphology. They received their name due to the involvement in the septum formation during yeast budding and are expressed in all eukaryotes, except in plants (Hartwell et al., 1974; Kinoshita and Noda, 2001; Pan et al., 2007). The first human septin was described in 1994 (Nakatsuru et al., 1994). To date, 13 functional septin genes encoding tissue-specific and ubiquitous expressed septins (*SEPT1* to *SEPT12* and *SEPT14*) have been identified (Kinoshita, 2003a). The number of septins varies between eukaryotic cells, from one in some algae to 13 in humans (Cao et al., 2009; Nishihama et al., 2011). Septins are an evolutionarily highly conserved protein group and belong to the Ras-like GTPase superclass of phosphate-binding loop (P-loop) NTPases (Leipe et al., 2002). All known septins (30–65 kDa) share a common structural domain organization (Figure 1A): A highly conserved central GTP-binding region, a variable amino-terminus, and a carboxyl-terminus often predicted to form coiled-coil structures, possibly involved in mediating protein-protein interactions (Trimble, 1999). Between the N-terminus and the GTPase domain, a short polybasic region (PBR) is found in most septin sequences, which binds directly to phosphatidylinositol bisphosphate (PIP_2_), and may be responsible for mediating interactions with plasma membranes (Casamayor and Snyder, 2003). A septin-unique domain (SUD) of unknown function exists C-terminally (Pan et al., 2007). The GTPase domain (G-domain) itself has a mixed α-helix/β-sheet secondary-structure and contains three motifs: G1 (GxxxxGK[s/T]) is characterized by the presence of the P-loop and is capable of interacting with nucleotide phosphate groups. Both G3 (DxxG) and G4 (xKxD) are directly associated with GTP binding (Sirajuddin et al., 2009). Based on their sequence homology and the number of coiled-coil domains, mammalian septins can be subdivided into four different groups termed according to their founding member SEPT2, SEPT3, SEPT6, or SEPT7 (Figure 1B). The SEPT2 subgroup (SEPT1, 2, 4, 5) contains two coiled-coil domains. The SEPT3 subgroup (SEPT3, 9, 12) has no coiled-coil domain. The SEPT6 (SEPT6, 8, 10, 11, 14) and the SEPT7 subgroup (SEPT7), respectively comprise one coiled-coil domain (Macara et al., 2002; Kinoshita, 2003b).

## Specificity of septin interactions

Unlike RAS-like GTP-binding proteins the septins can assemble into multimeric complexes including two or more subunits depending on the organism (Field et al., 1996; John et al., 2007; Sellin et al., 2011a). The classification of mammalian septins into four subgroups has relevance for the formation of these septin complexes because individual septins from each group can polymerize into a variety of higher-order structures, such as filaments, bundles, or rings (Mostowy and Cossart, 2012). Several studies have shown subgroup-restricted binding preferences of mammalian septins (Table 1) and that the typical filamentous form involves a hetero-trimer as its core module (Sirajuddin et al., 2007; Nakahira et al., 2010; Sandrock et al., 2011; Sellin et al., 2011a). Sirajuddin et al. first solved the crystal structure of the mammalian septin complex containing SEPT2, 6, and 7 and demonstrated its hexameric composition with mirroring symmetry arranged as a head-to-head trimer in the order 7-6-2-2-6-7 (Sirajuddin et al., 2007) (Figure 1C). In humans septins self-assemble predominantly into hetero-hexamers or hetero-octamers, whereas the hetero-octamers additionally contain SEPT3 subgroup members (Sellin et al., 2014). Sandrock et al. and Kim et al. showed that SEPT9 caps the ends of octameric complexes with SEPT7 (9-7-6-2-2-6-7-9) (Kim et al., 2011; Sandrock et al., 2011) (Figure 1D).

**Table 1:** **Subgroup-specific septin-septin binding preferences, septin-interacting proteins, and their physiological relevance**.

**Septin subgroup**	**Preferred partner**		**References**
SEPT6 group	SEPT2 group, SEPT3 group, SEPT7 group		Nakahira et al., 2010; Sandrock et al., 2011
SEPT2 group	SEPT6 group		Nakahira et al., 2010; Sandrock et al., 2011
SEPT3 group	SEPT7 group		Nakahira et al., 2010; Sandrock et al., 2011
**Protein**	**Interacting septin**	**Physiological relevance**	**References**
CENP	Septin 1, 2, 4, 5, 7, 9	Exocytose, intracellular trafficking	Nakahira et al., 2010
SNX6	Septin 2, 5, 6, 8, 11	Exocytose, intracellular trafficking	Nakahira et al., 2010
Sec6/8	Septin 2, 4, 6, 7	Vesicle transport	Ihara et al., 2005
Syntaxin1A	Sepin 2, 5	Vesicle transport	Beites et al., 2001
VAMP1	Septin 4, 11	Vesicle transport	Zhang et al., 2000
Transferrin receptor	Septin 4, 11	Vesicle transport	Zhang et al., 2000
FLNA	Septin 9	Cytoskeleton organization, vesicle transport	Nakahira et al., 2010
SH3KBP1	Septin 9	Cytoskeleton organization, vesicle transport	Nakahira et al., 2010
IFT27	Septin 3, 7	Vesicle transport, endocytose	Nakahira et al., 2010
Ra1ABP1	Septin 3, 7	Vesicle transport, endocytose	Nakahira et al., 2010
Actin cytoskeleton	Septins	Multiple functions	Kinoshita et al., 2002; Spiliotis et al., 2005
Microtuble cytokeleton	Septins	Multiple functions	Surka et al., 2002; Nagata et al., 2003; Kremer et al., 2005; Joo et al., 2007; Bowen et al., 2011; Mostowy and Cossart, 2012
Pospholipid membrane	Septins	Multiple functions	Spiliotis et al., 2008; Tanaka-Takiguchi et al., 2009
Anillin	Septin 2	Filament organization	Spiliotis et al., 2005
α-Tubulin	Septin complexes	Regulation of actin and tubulin polymerization	Nagata et al., 2003
Cytochalasin D	Septin 2, 6	Regulation of actin polymerization	Spiliotis et al., 2005
Latrunculin	Septin 2, 6	Regulation of actin polymerization	Spiliotis et al., 2005
MAP4	Septin 2-6-7 complex	Modulation of microtuble dynamics	Bowen et al., 2011
HDAC6	Septin 7	Dendritic development	Ghossoub et al., 2013
ERK3	Septin 7	Dendritic morphology	Ageta-Ishihara et al., 2013
Aurora-B	Septin 1	Mitose, cytokinesis	Hu et al., 2012
Cdk1/Pin1	Septin 9	Cytokinesis	Qi et al., 2005
Drp1	Septin 2	Mitochondrial fission	Estey et al., 2013
Tau, NFTs	Septin 1, 2, 4	Neuronal differentation and growth	Pagliuso et al., 2016
Parkin	Septin 5	Regulation of neuronal differentation and growth	Takehashi et al., 2004
α-Synuclein	Septin 4	Regulation of neuronal differentation and growth	Dong et al., 2003; Ihara et al., 2003
MLL	Septin 5, 6, 9, 11	Myeloid-lymphoid leukemia	Megonigal et al., 1998; Zieger et al., 2000
BORG3-cdc42	Septin 6, 7	Cell polarity, cytokinesis, vesicle transport	Engidawork et al., 2003; Kuo et al., 2015
BORG4-AP-3	Septin 6, 7	Regulation of endocytose	Joberty et al., 2001; Baust et al., 2008
KIF17	Septin 9	Intracellular protein transport	Nakahira et al., 2010; Traikov et al., 2014
UBE21, SUMO, PIAS	Near all septins	Protein degradation	Nakahira et al., 2010

The mammalian six subunit core heteromers are apparently stable protein complexes (Sellin et al, 2011b) but several studies showed that within one septin subgroup the individual members can substitute for one of the others at the same position of the complex *in vivo* and *in vitro* (Nakahira et al., 2010; Sandrock et al., 2011). For example, SEPT2 in the SEPT2-6-7 complex is replaceable by SEPT4 or 5 (Figure 1E), SEPT6 by SEPT8, 10, or 11. Several different trimers have been described, including SEPT3-5-7 (Fujishima et al., 2007), SEPT4-5-8 (Martinez et al., 2004), SEPT5-7-11 (Xie et al., 2007), SEPT7-11-9b (Nagata et al., 2004). The subunit heteromers depend on SEPT7 for stability (Sellin et al., 2011a). Mutations of a potential phosphorylation site within SEPT7 regulates the binding to all other septins (Sandrock et al., 2011). The higher-order septin structures are assembled from a mixture of hexamers and octamers, which all include the SEPT7 and variable SEPT2, 3, and 6 subgroup members. The composition of the septin complex is cell-type specific and essential for certain functions. At the mammalian sperm annulus SEPT12 as well as SEPT9 can flank the SEPT2-6-7 hexamers to form octamers (e.g., 12-7-6-2-2-6-7-12 or 12-7-6-4-4-6-7-12), suggesting a critical role in sperm motility (Kuo et al., 2015). These interaction studies in yeast and immunoprecipitation approaches have hinted toward the existence of diverse non-canonical septin complexes, but future studies are needed to determine the factors determining heteropolymer assembly.

Septins polymerize and interact with other septins via two interaction interfaces (G- and NC-interface) (Sirajuddin et al., 2007, 2009). The G-interfaces comprise the GTP-binding domain whereas the NC-interfaces involve the N- and C-terminal regions, which are brought into close proximity upon folding (Sheffield et al., 2003). This means, each septin subunit assembles and extends apolar filaments arranged in a palindromic order by alternating NC- and G-interface associations (Figure 1C). Upon GTP-binding a conformational change in the switch regions is provoked, which affects the G- and the NC-interface. The GTP-binding capacity of septins is essential for septin-septin interactions and fundamental to ensure structural integrity, in a way that GTP-binding and its hydrolysis control assembly and disassembly of filaments and also the stability of the interface within the septin polymer (Zeraik et al., 2014). In the SEPT2-6-7 complex SEPT2 and 7 bind to GDP, while SEPT6 binds to GTP (de Almeida Marques et al., 2012). A Sept11 mutant, which showed reduced GTPase activity, was unable to form filaments (Hanai et al., 2004). Moreover, the GTPase domain seems to form homo-dimers and homo-filaments *in vitro* (Garcia et al., 2006; Huang et al., 2006; Nakahira et al., 2010). Studies showed that single septins may be unstable and assemble into homo-dimers or a fibrillary aggregated form called amyloid in the absence of GTP and by unbalanced stoichiometries. Depending on the temperature SEPT2 can exist as a dimer and contains regions within its G-domain sequence with a tendency to aggregate and/or form amyloids (Pissuti Damalio et al., 2012). Thereby, a decrease of α-helical content and a gain in β-sheet structure has been observed. Also homo-multimers have been reported for human SEPT2 in both the GTP- and GDP-bound states (Huang et al., 2006). Furthermore, Garcia et al. reported that SEPT4-G (an intermediate structure of the GTPase domain of human SEPT4) can form homo-filaments and amyloid-like aggregates (Garcia et al., 2007, 2008).

Due to alternative mRNA splicing many human septin genes present several variants. Cell culture models showed that the human SEPT9 exists as multiple isoforms, which have a common G-domain but differ in both length and sequence of the N-terminus. The SEPT9 isoform and expression level determine the higher-order arrangement of septin filaments (Sellin et al., 2012). In cells lacking SEPT7 mutational analysis of interaction surfaces reveals that SEPT9 exists as monomer (Kim et al., 2011; Sellin et al., 2011a). Besides the G-domain, the C-terminal domains and their coiled-coil regions are important determinants for filament assembly and stability and are important for recognition and binding of partner molecules (de Almeida Marques et al., 2012). For example, some septins interact with proteins (CENP-E/F, SNX6), which are associated with intracellular trafficking and exocytosis or are part of the kinetochore via coiled-coil domains (Nakahira et al., 2010) (Table 1). Filaments containing septins are implicated in exocytosis and are closely involved in membrane transport and fusion (Blaser et al., 2004; Ihara et al., 2005). Septins may associate to provide a targeting system to recruit secretory vesicles to appropriate docking/fusion sites leading to the correct organization of proteins along the plasma membrane. In brain lysates of rats the septins SEPT2, 4, 6, and 7 are associated with the exocyst complex sec6/8 that is essential for neuronal vesicle transport (Hsu et al., 1998). In neurons SEPT2 and 5 have been shown to interact with syntaxin 1A, a t-SNARE protein predominantly present on the plasma membrane, and they copurify with synaptic vesicles (Beites et al., 1999, 2001; Zhang et al., 2000). Bartsch et al. demonstrated the colocalization of SEPT4 and 11 with the vesicle-associated protein synaptobrevin 1 (VAMP1) and the endocytotic transferrin receptor (Bartsch et al., 2010). Moreover, SEPT9 interacts with filamin A (FLNA) and SH3-domain kinase binding protein 1 (SH3KBP1) via N-terminus (Nakahira et al., 2010), two proteins involved in cytoskeleton organization and vesicle transport (van der Flier and Sonnenberg, 2001; Spiliotis et al., 2005). Further septin partners associated with vesicle transport are other Ras-like GTPases (IFT27, Ra1ABP1) (Nakahira et al., 2010).

Mammalian septins interact with actin (Kinoshita et al., 2002; Joo et al., 2007) and microtubule cytoskeletons (Surka et al., 2002; Nagata et al., 2003; Kremer et al., 2005; Spiliotis et al., 2008; Bowen et al., 2011; Sellin et al, 2011b), as well as with phospholipid membranes (Tanaka-Takiguchi et al., 2009; Bertin et al., 2010). Thus, they assemble at specific locations in the cell to coordinate changes in membrane and cytoskeletal organization by acting as cell scaffolds for protein recruitment to specific sites in a cell and/or as lateral diffusion barriers in the plasma membrane to compartmentalize discrete cellular domains. Unlike actin and microtubules, septin complexes are apolar along the longitudinal axis in recombinant systems. One important actin-binding protein, which interacts with septins at the cell surface is anillin (Kinoshita et al., 2002). Anillin mediates the septin filament organization along actin bundles by recruiting and stabilizing myosin, actin, and regulatory kinases (Joo et al., 2007; Maddox et al., 2007). Septin complexes interact with α-tubulin and control actin and tubulin polymers (Surka et al., 2002). In fibroblasts cytochalasin D or latrunculin inhibit the actin polymerization and the septin filaments disappear while septin rings occur, which are not associated with actin (Kinoshita et al., 2002). The microtubule-associated protein MAP4, which is required for modulation of microtubule dynamics during mitosis and cytokinesis, is another septin binding partner. MAP4 is recruited to the SEPT2-6-7 complex via the direct interaction of its C-terminal proline-rich domain with SEPT2 (Kremer et al., 2005). SEPT2 together with MAP4 is involved in the organization of primary cilia (Ghossoub et al., 2013). In addition, SEPT7 is associated with transport of organelles and regulates dendritic morphology (Xie et al., 2007) by interaction with α-tubulin deacetylase HDAC6 (Ageta-Ishihara et al., 2013) or extracellular signal-regulated kinase 3 (ERK3) (Brand et al., 2012). In developing axon collateral branches, SEPT7 influences the remodeling of microtubules into filopodia, a process required for successful formation of branches (Hu et al., 2012). Tubulin-associated SEPT2 facilitates vesicle transport from the Golgi to the plasma membrane (Spiliotis et al., 2008). Furthermore, septins are involved in cell division. SEPT1 is a target of aurora-B, which is an important serine/threonine kinase required for chromosome segregation and cytokinesis (Qi et al., 2005). Phosphorylation of SEPT9 by cyclin-dependent kinase 1 (Cdk1) regulates association with the proline isomerase (Pin1), which is crucial for the disjunction of daughter cells (Estey et al., 2013).

Recently SEPT2 has been shown to participate in dynamin-like protein Drp1-dependent mitochondrial fission (Pagliuso et al., 2016). Misregulation of human septins is associated with numerous diseases, like neurodegenerative disorders. For example, septins are involved in Alzheimer's disease (AD) because they interact with neurofibrillary tangles (NFTs). SEPT1, 2 and 4 bind the microtubule-associated protein tau, a major component of the neurofibrillary tangles, which is important for neuronal differentiation and growth (Kinoshita et al., 1998). Furthermore, genotype studies of polymorphic SEPT3 alleles in human neuronal cells indicated a significant difference between AD patients and controls (Takehashi et al., 2004). Another binding partner of septins is parkin, which is mutated in autosomal-recessive juvenile parkinsonism (ARJP) (Kitada et al., 1998). Parkin, an ubiquitin ligase, interacts with SEPT5 and mediates its degeneration (Choi et al., 2003; Son et al., 2005). A loss of parkin causes accumulation of SEPT5 in neurons of patients with ARJP and induces selective dopamine-dependent neurodegeneration. SEPT5 inhibits the release of dopamine (Zhang et al., 2000; Choi et al., 2003; Dong et al., 2003; Son et al., 2005). In Parkinson‘s disease or dementia SEPT4 is accumulated in cytoplasmic aggregates colocalizing with the dopamine receptor α-synuclein. Sept4 deficient mice exhibit diminished dopaminergic neurotransmission due to the lack of SEPT4 (Ihara et al., 2003, 2005, 2007). SEPT4 is a distinct gene product with a >90% identity to SEPT5 (Zieger et al., 2000). Some human septin proteins (SEPT5, 6, 9, and 11) have been cloned as fusion partner of myeloid-lymphoid leukemia *MLL* (also referred to as *ALL1* or *HRX*) genes. These fusion proteins consist of almost the entire open reading frame of the involved septin and the N-terminus of MLL (Megonigal et al., 1998; Kojima et al., 2004). Thus, misregulation of human septins plays a role in cancer. SEPT9 supports HIF-1α-mediated transcription in tumor cells (Amir et al., 2009), suggesting that SEPT9 is a player in posttranslational modification. Down-syndrome patients showed an increased expression of mixed lineage leukemia septin like fusion protein (MSF)-B, suggesting a hint why Down-syndrome children show a stringent incidence of acute leukemia (Engidawork et al., 2003).

Septins have been shown to interact with BORGs (binding partners of RHO-GTPases; CDC42 effector proteins). For example, BORG3 binds directly to SEPT6 or 7 (Joberty et al., 2001; Sheffield et al., 2003). BORGs (BORG1 to BORG3) interact generally with two types of GTPases, namely septins and CDC42. Overexpression of constitutively active CDC42-GTPase markedly affects the association of BORG3 with septins and disrupts normal septin complex organization leading to a pathological localization of the septins within the cell. Thus, BORGs are important regulators of mammalian septin organization and provide a link between the septins and CDC42-GTPases, which regulate cell polarity, cytokinesis, cytoskeletal remodeling and vesicle transport (Joberty et al., 2001). Baust et al. identified a septin-/BORG-protein network und hypothesized that BORG4 and septins are important regulators for the AP-3 adaptor complex-dependent sorting of the lysosome membrane protein 1 (LAMP-1) to lysosomes (Baust et al., 2008). AP-3 is a member of an adaptor complex, which is involved in the targeting of cargo destined to remain in outer membranes of maturing endosomal compartments. In this complex, SEPT6 and 7 function as regulators of endosome transport by modulating the timely coordinated interaction of AP-3 (Traikov et al., 2014). In addition, SEPT9 interacts with the kinesin 2 family motor KIF17, which is a cargo/scaffold protein, suggesting the importance of SEPT9 in transport mechanisms in neurons (Bai et al., 2016). SEPT9 also regulates growth and accumulation of lipid droplets, which are frequently observed in hepatitis C virus infection (HCV), by a phosphatidylinositol-5-phosphate and microtubule-dependent binding mechanism in HCV-infected cells (Akil et al., 2016).

Nakahira et al. described septin interactions with several proteins that play a role in protein degeneration and functionally associated with the ubiquitin and sumoylation cycles (e.g., UBE21, SUMO, or PIAS) (Nakahira et al., 2010). Sumoylation generally controls assembly, localization, stability and other functions of protein complexes (Schmidt and Muller, 2003; Johnson, 2004).

## Septin interactions in platelets and human endothelial cells

In platelets several septins (SEPT2, 4, 5, 7, 8, 9, and 11) are expressed, which seem to be important for regulating platelet function (Yagi et al., 1998; Zieger et al., 2000; Blaser et al., 2003; Bartsch et al., 2010; Sandrock et al., 2011). Transmission electron microscopy revealed that SEPT4 and 8 surround the α-granules, as it had been shown for SEPT5, suggesting that they may be components of the same complex in platelets and play in such a way a general regulatory role in platelet biology. Activation of platelets by agonists resulted in the translocation of SEPT4 and 8 to the platelet surface indicating a possible functional role of these proteins in platelet granular transport and secretion (Dent et al., 2002; Blaser et al., 2004; Martinez et al., 2006). Platelets from Sept5 deficient mice showed altered granule release (serotonin) and revealed a decreased threshold for agonist-mediated platelet aggregation, suggesting that SEPT5 is involved in platelet physiology (Dent et al., 2002). Sept5 deficient mice presented a marked increase in bleeding symptoms compared to wild-type mice. The regulatory factors promoting vesicle docking and fusion after platelet stimulation are unknown so far, but it might be a large multimeric complex of various proteins involved in platelet biology. The interaction in a macromolecular complex in platelets between SEPT5 and syntaxin-4, which is involved in vesicle transport, suggests a cooperation of these proteins supporting the critical role of SEPT5 in granule secretion. Interestingly, in platelets SEPT5 has a strong affinity for other septins, such as SEPT4 and 8, which are expressed together in various tissues (Blaser et al., 2002; Martinez et al., 2004). In addition, SEPT5 has been shown to be colocalized with SEPT6 in the periphery of the platelet and in the cytoplasma and was associated with platelet microtubules in the SEPT5-6-7 complex promoting the important role of septins in granule trafficking through their association with the microtubule network. Martinez et al. revealed furthermore that SEPT9 is part of the SEPT5-6-7-9 complex in platelets (Martinez et al., 2006). In platelets and HUVECs, SEPT5 has been identified as an interaction partner of SEPT11, which may also be involved in regulated secretion (Blaser et al., 2006). Many septins are expressed ubiquitously, for example SEPT6 (Ono et al., 2002), SEPT7, 9, and 11 (Hanai et al., 2004) while a subset appears tissue restricted (Hall et al., 2005; Cao et al., 2007), like SEPT5 (previous: hCDCrel-1; human cell division cycle gene, PNUTL1) (Macara et al., 2002), which is expressed predominantly in platelets, brain, and heart (McKie et al., 1997; Yagi et al., 1998). In addition, SEPT5 deficiency exerts pleiotropic effects on affective behaviors and cognitive functions as shown in Sept5 knock-out mice, which feature delayed acquisition of rewarded goal approach (Suzuki et al., 2009). In HIT-T15 cells mutations in SEPT5 inside the GTP-binding domain lead to an increased granule secretion (Beites et al., 1999). As shown before, in human endothelial cells SEPT4 and 11 are involved in endo- and exocytotic processes by interacting with vesicle-associated proteins (Bartsch et al., 2010). These studies confirm the binding preferences of the SEPT2 subgroup (SEPT4 and 5) with members of the SEPT6 subgroup (SEPT8 and 11) (Figure 1E).

Interestingly, mice with Bernard-Soulier-syndrome (BBS) caused by genetic deletion of the platelet glycoprotein (GP) Ibβ (GP1BB) demonstrated increased levels of SEPT5 in the megakaryocytic linage. Overexpression of SEPT5 is associated with fewer and larger platelet α-granules, suggesting that SEPT5 supports normal α-granule size in wild-type littermates (Kato et al., 2004). *SEPT5* was identified as a 5′gene located in close 5′proximity to the *GPIb*β gene (Zieger et al., 1997). GPIbβ is besides GPIbα a subunit of GPIb, which is a major component of the platelet membrane receptor (GPIb/IX) for von Willebrand factor (Ruggeri, 1991). Both genes, *SEPT5* and *GPIb*β, are located within the chromosomal locus 22q11.2, a region associated with the DiGeorge syndrome (DGS) and other cardio-facial abnormalities (McDermid and Morrow, 2002). The close relationship of DGS and GPIbβ has been confirmed by chromosomal deletion of 22q11.2 in a patient with both DGS and BSS (congenital absence of the platelet GPIb/V/IX receptor complex) (Budarf et al, 1995).

In a boy with a unique homozygous deletion of the two contiguous genes *SEPT5* and *GPIb*β resulted in a BSS phenotype (reduced expression of the GPIb/V/IX receptor) combined with a platelet secretion defect (Bartsch et al., 2011). The patient suffered from life-threatening bleedings, which could be hardly stopped. Therefore, he received hematopoietic stem cell transplantation. Because of the deletion of SEPT5 (which is also important for vesicle transport and exocytosis in neurons) the patient showed an additional secretion defect of platelet α-granules. Most probably, the platelet secretion defect resulted in an additive effect regarding his bleeding symptoms besides the BSS defect. Furthermore, the patient is retarded in development and shows autistic streaks. The cortical dysplasia (polymicrogyry) and neurologic dysfunction is likely caused by the SEPT5 deletion. His parents both exhibit a heterozygous deletion on this area.

## Conclusion

Diverse studies showed cell-specific formation of mammalian septin-multimers and their association with a variety of the cellular processes, such as actin dynamics, microtubule regulation, membrane trafficking, vesicle transport, exocytosis, the assembly of scaffolding platforms, protein degradation, and mechanical stability. Many septins have been associated with diverse human diseases, such as neurodegenerative and bleeding disorders. Homozygous deletion of ubiquitary septins results in embryonic lethality (Roseler et al., 2011). Exact structural properties and many of the molecular details and modes of action remain unclear. The complex relationship between polymerization, bundling, GTPase activity and membrane association needs to be elucidated in future studies.

## Author contributions

All authors listed, have made substantial, direct and intellectual contribution to the work, and approved it for publication.

## Funding

This work was supported by a grant from Deutsche Forschungsgemeinschaft (ZI 486/4-1).

### Conflict of interest statement

The authors declare that the research was conducted in the absence of any commercial or financial relationships that could be construed as a potential conflict of interest.
